# The impact of harm reduction on HIV and illicit drug use

**DOI:** 10.1186/1477-7517-11-7

**Published:** 2014-02-21

**Authors:** Lianping Ti, Thomas Kerr

**Affiliations:** 1 British Columbia Centre for Excellence in HIV/AIDS, St. Paul’s Hospital, 608 - 1081 Burrard Street, Vancouver, BC V6Z 1Y6, Canada; 2 School of Population and Public Health, University of British Columbia, Vancouver, BC V6T 1Z3, Canada; 3 Department of Medicine, University of British Columbia, St. Paul’s Hospital, Vancouver, BC V6Z 1Y6, Canada

**Keywords:** Harm reduction, Illicit drug use, Canada

## Abstract

There has been widespread support for harm reduction programs as an essential component for responding to the HIV and illicit drug use epidemics. However, despite the growing international acceptance of harm reduction, there continues to be strong opposition to this approach, with critics alleging that harm reduction programs enable drug use. Vancouver, Canada provides a compelling case study that demonstrates that many positive impacts of harm reduction can be attained while addiction treatment-related goals are simultaneously supported. While the evidence for harm reduction is clearly mounting, it is unfortunate that ideological and political barriers to implementing harm reduction programs in Canada remain. As evidenced by Vancouver and elsewhere, harm reduction programs do not exacerbate drug use and undermine treatment efforts and should thereby occupy a well-deserved space within the continuum of programs and services offered to people who inject drugs.

## Commentary

### Background

The widespread support for harm reduction programs as essential responses to the harms of illicit drug use continues to grow [1]. International health bodies, including the World Health Organization (WHO) and the Joint United Nations Programme on HIV/AIDS (UNAIDS), recommend harm reduction programs as best practices and crucial for reducing HIV infection among people who inject drugs (IDU) [1]. The WHO/UNAIDS comprehensive HIV prevention package for the prevention, treatment, and care of HIV among IDU recommends the provision of sterile needles and syringes as well as opioid substitution therapy, and in response, public health and nongovernmental organizations in various settings have rolled out these programs [2,3].

However, despite the growing international acceptance of harm reduction approaches as an evidence-based strategy for minimizing the negative consequences related to illicit drug use, opposition to harm reduction persists. Those strongly opposed to harm reduction typically argue that programs such as syringe distribution and supervised injecting facilities (SIFs) enable drug use and undermine drug treatment efforts [4]. However, much of these arguments have relied on unpublished reports by anti-drug lobby groups and have been deemed questionable for failing to meet accepted academic standards [5,6].

However, as such criticisms and concern continue to be aired and repeated by some media outlets [7], the evidence in support of harm reduction continues to grow, as does the body of research demonstrating that harm reduction does not enable drug use at the individual nor the community level. The city of Vancouver (Canada) provides an interesting case example of such effects. In the late 1990s, Vancouver was the site of massive epidemics of HIV infection and overdose among IDU. In response, the regional health authority launched an aggressive public health response, which included scaling up syringe distribution, peer-based programming, methadone maintenance therapy, and establishing the first North American sanctioned SIF. A recent report examining data derived from three US National Institute of Drug Abuse-funded cohort studies revealed that rates of HIV and HCV infection as well as other indicators of drug-related risks and harms have plummeted in Vancouver over the past 15 years [8]. For example, syringe sharing has been reduced from approximately 40% in 1996 to less than 2% in 2011, and this can be largely attributed to an increase in the distribution of sterile injecting paraphernalia [8]. At the same time, there has been a dramatic increase in drug injection cessation among IDU in this setting. As the report also shows, these health gains have been made despite the failure of drug supply reduction efforts, evidenced by the fact that the accessibility and price of illicit drugs have remained stable in Vancouver for over 10 years [8].

While it is clear that local policy makers in Vancouver have been convinced of the value of harm reduction programs, sadly, it appears that the federal conservative government of Canada has chosen to ignore evidence and embrace a drug policy approach that is not only costly but also ineffective in reducing illicit drug use and supply [8,9]. Such unsuccessful policies continue to place undue harm upon IDU by placing an emphasis on failed law enforcement approaches in combatting illicit drug use [10,11]. Further, for the first time since 1987, the words ‘harm reduction’ have been removed from Canada’s national drug strategy [12]. The federal government has also remained opposed to Insite, Vancouver’s SIF. However, despite their numerous attempts to shut the facility down, a unanimous 9-0 Supreme Court ruling has allowed this life-saving program to continue to operate under an exemption from federal drug laws [13]. Still, the federal government continues to set unnecessary roadblocks in scaling up SIFs in Canada. The introduction of Bill C-2, known as the Respect for Communities Act, requires local community and police support before a new SIF can be implemented and gives the federal Minister of Health sole authority in approving new SIFs to operate under the exemption [14]. In essence, the government is putting ‘NIMBYism’ and policing interests ahead of public health. If this new legislation is reintroduced and passed, applicants will face significant obstacles in attempting to open SIFs across Canada. This could have the effect of preventing IDU from accessing low-barrier health services and will thereby threaten the health and lives of some of Canada’s most vulnerable citizens.

The evidence in support of harm reduction programs only continues to grow, as does the evidence showing that harm reduction programs do not exacerbate individual and community drug use patterns. It is clear that programs like Insite save lives and support rather than undermine treatment efforts by connecting individuals to various forms of addiction treatment. Sadly, despite the evidence from Vancouver showing that the harm reduction response served to significantly reduce drug-related harms without increasing drug use locally, barriers to implementing harm reduction programs in Canada remain. These barriers, all social and political in nature, have immense potential to exacerbate preventable human suffering and place a massive and unnecessary burden on the Canadian healthcare system.

### Conclusion

The time to heed the recommendations of the world’s leading health bodies has come. Tired arguments against harm reduction persist, but these come from those who ignore evidence and put ideology and politics ahead of public health. It can no longer be argued, in a compelling fashion, that harm reduction exacerbates drug use and undermines treatment efforts. The evidence from Vancouver and elsewhere clearly shows that harm reduction programs typically do what they are designed to do - they reduce drug-related harm, support addiction treatment efforts, and thereby occupy a well-deserved space with the continuum of programs and services offered to IDU.

## Competing interests

The authors declare that they have no competing interests.

## Authors’ contributions

LT and TK developed the original arguments and completed the first and final drafts of the manuscript. Both authors read and approved the final manuscript.
